# Predictive Factors of the Response of Rectal Cancer to Neoadjuvant Radiochemotherapy

**DOI:** 10.3390/cancers3022176

**Published:** 2011-04-26

**Authors:** Gaya Spolverato, Salvatore Pucciarelli, Roberta Bertorelle, Anita De Rossi, Donato Nitti

**Affiliations:** 1 Department of Oncology and Surgical Sciences, Section of Surgery, University of Padova, Padova 35128, Italy; E-Mails: gaya_spolverato@yahoo.it (G.S.); puc@unipd.it (S.P.); donato.nitti@unipd.it (D.N.); 2 Istituto Oncologico Veneto-IRCCS, Padova 35128, Italy; E-Mail: roberta.bertorelle@unipd.it (R.B.); 3 Department of Oncology and Surgical Sciences, Section of Oncology, University of Padova, Padova 35128, Italy

**Keywords:** rectal cancer, neoadjuvant therapy, biomarkers

## Abstract

Locally advanced rectal cancer is currently treated with pre-operative radiochemotherapy (pRCT), but the response is not uniform. Identification of patients with higher likelihood of responding to pRCT is clinically relevant, as patients with resistant tumors could be spared exposure to radiation or DNA-damaging drugs that are associated with adverse side effects. To highlight predictive biomarkers of response to pRCT, a systematic search of PubMed was conducted with a combination of the following terms: “rectal”, “predictive”, “radiochemotherapy”, “neoadjuvant”, “response” and “biomarkers”. Genetic polymorphisms in epithelial growth factor receptor (EGFR) and thymidylate synthase (TS) genes, the expression of several markers, such as EGFR, bcl-2/bax and cyclooxygenase (COX)-2, and circulating biomarkers, such as serum carcinoembryonic antigen (CEA) level, are promising as predictor markers, but need to be further evaluated. The majority of the studies did not support the predictive value of p53, while the values of Ki-67, TS and p21 is still controversial. Gene expression profiles of thousands of genes using microarrays, microRNA studies and the search for new circulating molecules, such as human telomerase reverse transcriptase mRNA and cell-free DNA, are providing interesting results that might lead to the identification of new useful biomarkers. Evaluation of biomarkers in larger, prospective trials are required to guide therapeutic strategies.

## 1. Introduction

Colorectal cancer (CRC) is the third most common cancer in the world, with an estimated 72,090 new cases/year for males and 70,480 for females in the USA. It is also the third most common cause of cancer-related death, with an estimated 26,580 and 24,790 deaths/year for males and females, respectively [1]. The estimated incidence in Europe and Italy respectively is 333,330/51,686 with a mortality of 148,788/19,103 [2]. Approximately 20% of CRC diagnoses are distal to the recto-sigmoid junction and are designated as rectal cancer [3].

CRC is the result of genetic and epigenetic alterations that cause disorders in cell growth, differentiation and apoptosis, resulting in transformation of normal epithelium to adenocarcinoma [4]. The accumulation of molecular alterations in oncogenes (e.g., KRAS) and loss of oncosuppressor genes (e.g., APC, TP53) occurs with stepwise changes in morphology from a small adenomatous polyp to an invasive carcinoma [5-7].

Pre-operative radiochemotherapy (pRCT) is currently the standard treatment for locally advanced rectal cancer (clinical TNM stage II—III) [8]. Using this approach, the outcomes are encouraging, with 5-year rates of local and distant recurrence ranging from 6% to 9% and 33% to 36%, respectively [9-11]. However, the rates of chemotherapy-, radiotherapy- and surgery-related toxicity, as well as bowel and sexual dysfunction, are very unsatisfactory [12]. The peculiar aspects of this approach are related to clinical overstaging, which may result in an unnecessary neoadjuvant treatment in almost one-fifth of patients [10]. Approximately 40% of patients show poor or no response to pRCT, while a pathologic complete response (pCR) has been reported in up to 44% of cases [13,14]. For patients with a pCR, less aggressive approaches have been advocated [15], such as the “wait and see” policy [16] or transanal local excision [17].

It is clinically relevant to identify predictive markers of cancer response to different combinations of treatments. Prior identification of patients who have a higher likelihood of responding to pRCT could help to select those who can benefit from the treatment. Patients with a known resistant tumor could be spared exposure to radiation or DNA-damaging drugs that are associated with adverse side effects. Several biomarkers have been correlated with clinical staging and outcome; they are categorized as prognostic and predictive. Predictive markers are related to the impact of the treatment on the outcome, while prognostic markers are related to the outcome independent of treatment [4]. These considerations have prompted researchers to find biomarkers that can predict the tumor response both before and after neoadjuvant treatment.

The aim of this review is to examine the most common molecular markers analyzed as predictors of the response to pRCT in patients with rectal cancer.

## 2. Materials and Methods

A systematic search of PubMed was conducted with a combination of the following terms: “rectal”, “predictive”, “radiochemotherapy”, “neoadjuvant”, “biomarkers” and “response”. The range of time chosen was between 2005 and 2010. Articles were hand-searched for those of relevance. Those including patients who had received single-modality therapy or adjuvant but not neoadjuvant treatment were excluded. The only studies included were those in which the patients affected by rectal cancer were treated with pRCT. Fifty-eight articles that evaluated biological markers predicting the outcome of rectal cancer patients treated with pRCT were found.

The most commonly used endpoints to evaluate the response to pRCT are tumor regression grade (TRG), TNM downstaging, pCR and overall survival (OS). Mandard *et al.* [18] originally described TRG for oesophageal cancer, and later Dvorak *et al.* [19] adapted it for rectal cancer. Based on the ratio between fibrosis and residual tumor cells, the TRG classification stratifies tumor response into the following five classes:

TRG 1: complete response with absence of residual cancer and fibrosis;
TRG 2: presence of residual tumor cells scattered through the fibrosis;
TRG 3: increase in the number of residual cancer cells, with predominant fibrosis;
TRG 4: residual cancer outgrowing fibrosis;
TRG 5: absence of regressive changes.

TRG classification is used as the gold standard for evaluating tumor response to pRCT because it takes in account histopathological alterations caused by the treatment, namely radiotherapy [20]. Nevertheless, TRG does not provide any information about nodal metastasis or the depth of tumor invasion. TNM downstaging compares clinical TNM with pathological TNM staging [21]. Clinical TNM staging, as evaluated by magnetic resonance imaging (MRI), or computed tomography (CT) and/or trans-rectal ultrasound endoscopy (TRUS), is flawed by limits in the accuracy of these imaging modalities [22]. An even more controversial issue is the performance of the same imaging modalities to restage patients after pRCT and before surgery [23,24].

## 3. Results

In the articles reviewed, the biomarkers most frequently evaluated were p53, thymidylate synthase (TS), epidermal growth factor receptor (EGFR), Ki-67, p21 and bax/bcl-2. Other molecular markers cited at least in two articles were cyclooxygenase-2 (COX-2), cyclin D1, survivin, vascular endothelial growth factor (VEGF) and dihydropyrimidine dehydrogenase. Other biomarkers investigated for their potential predictive roles in the response to pRCT were microRNAs (miRNAs), microsatellite instability, mismatch repair proteins, gene expression profiles using microarray technology and the circulating level of carcinoembryonic antigen (CEA).

### 3.1. p53

p53 protein mediates cell cycle arrest and cell death checkpoints [25]. Inactivation of the p53 pathway is a secondary genetic step in CRC. This inactivation, due to allelic loss, gene mutation or protein sequestration, leads to increased genetic instability and survival of cells with damaged DNA [26]. p53 plays key roles in apoptosis, tumorigenesis and sensitivity to chemotherapeutic agents. Malignant cells with wild-type p53 are sensitive, whereas mutated p53 malignant cells are resistant to radiotherapy and chemotherapeutic agents [27-29].

A summary of studies performed using p53 expression as a predictor of response in patients with rectal cancer receiving pRCT is reported in Table 1. The majority of studies [30-38] did not find any association between p53 expression and treatment response. However, Lin *et al.* showed that absence of p53 protein in biopsy specimens obtained before radiation was a good predictor of poor response [39]. Although the role of p53 expression as a predictive factor for the response to pRCT is still controversial, it seems unlikely that it could be used as a marker of response.

### 3.2. Thymidylate Synthase

TS is the primary intracellular target of 5-fluoracil (5-FU), the most widely used chemotherapeutic agent for CRC [40]. TS is involved in DNA synthesis, playing a critical role in the phosphorylation of deoxyuridine monophosphate to deoxyuridine triphosphate. In CRC, the overexpression of TS is associated with 5-FU resistance [41].

In rectal cancer treated with pRCT, TS expression has been evaluated at the protein level by immunohistochemistry (IHC) and at the RNA level by polymerase chain reaction (PCR). In addition, genetic polymorphisms of the TS gene have been studied and related to TS expression and tumor response. A summary of studies performed on this topic is reported in Table 2. TS expression has been correlated with TRG: responders had low TS, whereas 75% of non-responders had high TS [36]. Moreover, TS level, along with Ki-67, has predictive value in rectal cancer treated with 5-FU-based pRCT, and the association with Ki-67 suggests that TS is involved in active cell cycle processes. However, Negri *et al.* [34] demonstrated that a high TS level is predictive of a higher pathological response. Carlomagno *et al.* [42] showed that rectal tumors with low TS expression had a 16.7-fold increased odds ratio of not obtaining a pCR. A high TS score tended to be associated with better pathological response in a multiple logistic regression analysis [43]. Conversely, another study [33], found no correlation between TS protein expression and response to pRCT.

Several studies analyzed genetic polymorphisms as potential predictive factors of the response to pRCT. In particular, three main polymorphisms have been studied. A 28-base pair tandem repeat (28-bp TR) of the TS enhancer region influences gene expression, with the number of repeats directly correlated with gene expression (3R *vs.* 2R alleles) [44]. However, a G > C single-nucleotide polymorphism (G > C SNP) in the second repeat of the 3R allele reduces TS mRNA expression [45]. Moreover, a 6-bp deletion localized in the 3′-UTR of the TS gene has been associated with decreased mRNA expression [46]. Stoehlmacher *et al.* [47] confirmed the association between the 3′-UTR 6-bp deletion and low TS mRNA expression and described a trend towards tumor response in patients receiving 5-FU-based pCRT, although the results were not statistically significant. Spindler *et al.* [48,49] studied in locally advanced T3 rectal tumous the polymorphism 28-bp TR: 8 of 15 (53%) patients with the TS 2R/2R genotype had a pCR compared to 10 of 45 (22%) patients with a TS 2R/3R or 3R/3R genotype (p = 0.048). Hur *et al.* [50] evaluated the 28-bp TR and found no significant difference in tumor response between patients with the 3R/3R and patients with the TS 2R/3R genotype; patients with 2R/2R were not present in the study. Moreover, 28-bp TR was considered along with the G > C SNP, and low-(2R/2R, 2R/3RC, 3RC/3RC) and high-expressing groups (2R/3RG, 3RC/3RG, 3RG/3RG) were defined. Thirteen of 14 patients in the low-expression genotype group exhibited a significantly greater tumor downstaging rate, as compared with 12 of 30 patients in the high-expression group. However, there was no significant difference in TRG between these groups. When considering the association between the presence of the high/low TS expression alleles and response to pRCT, Paez *et al.* [51] observed a higher response rate in patients with the 3R/3R TS genotype. Conversely, Terrazzino *et al.* [52] found no correlation between the 28-bp TR or the G > C SNP and response to pRCT.

Based on these findings, the association between TS expression or TS polymorphisms and tumor response after pRCT is still largely unclear. Among the several factors that can explain the variability of results, a relevant role might be played by the inclusion of oxaliplatin in the treatment schedules. In fact, even for tumors with high TS levels, oxaliplatin deregulates TS mRNA and TS protein expression, influencing the response to 5-FU [53].

### 3.3. Epidermal Growth Factor Receptor (EGFR)

EGFR mediates signaling by activating *via* KRAS, a small GTPase protein involved in signal transduction, the MAP kinase and PI3K cascades. It is involved in many cellular pathways, such as those driving proliferation, apoptosis and differentiation.

A summary of studies concerning its predictive role in tumor response to pRCT is reported in Table 3. In a trial of 183 patients with local advanced rectal cancer treated with pRCT, only patients with low EGFR expression had TNM downstaging [54]. Giralt *et al.* [55] showed that EGFR-positive tumors were associated with a decreased pCR rate and decreased disease-free survival. Toiyama *et al.* [56] found lower EGFR expression in TRG responders than in non-responders. Others [33,57] found no correlation between tumor response and EGFR expression.

EGFR-based chemotherapy is effective in the treatment of CRC, and some studies have been conducted by adding cetuximab to pre-operative chemoradiation regimens. Bengala *et al.* [58] indicate a trend for better response to cetuximab-based treatment in patients with a high gene copy number (GCN) of EGFR and wild-type *KRAS*. Notably, neither EGFR GCN nor KRAS status was correlated to TRG, when cetuximab was not included in the pRCT [59]. However, in other studies, pRCT with cetuximab gave disappointing results [60,61], and mutations in KRAS were not associated with response to cetuximab-based chemotherapy [62]. The low response rate observed might have been due to the strong antiproliferative effect of cetuximab, which may compromise the activity of chemotherapeutics that target proliferating cells [63].

Polymorphisms in the EGFR gene have been investigated as possible regulators of gene expression and consequently as predictive factors to pRCT. Spindler *et al.* [48] studied a SNP in the Sp1 binding site of the *EGFR* promoter (Sp1-216 G/T), which has been associated with higher gene expression, in a group of patients with locally advanced rectal cancer; patients with T variants had a better possibility of pCR than GG homozygous patients (65% for G/T and T/T *vs.* 34% for GG). Moreover, analyzing the combination of the TS 28-bp TR, the EGFR Sp1-216, and a polymorphism in the 5′UTR region of the EGF gene (SNP A61G; EGF61) revealed that a higher number of pCR was obtained in patients with TS 2R/2R and EGFR Sp1-216T or EGF61A/G than in patients carrying other polymorphism combinations (64% *versus* 21%) [48].

In conclusion, level of EGFR expression may be useful in the prediction of pathological response to pCRT; additional studies are needed to clarify the role of EGFR and KRAS in response to cetuximab-based neoadjuvant chemotherapy.

### 3.4. Ki-67

Ki-67 is a marker for cellular proliferation and can be detected in all active stages of the cell cycle [64]. Although the majority of studies did not find any association between Ki-67 expression and response to pRCT in rectal cancer [31,33,36,38,65-67], Jakob *et al.* [36] showed that rectal cancers treated with 5-FU with a low Ki-67 expression had a significantly better response than those with high Ki-67. As reported for TS, the multiple logistic regression analysis reported by Kikuchi *et al.* [43] revealed Ki-67 as an independent factor, with a sensitivity and specificity for prediction of response of 82.8 and 83.9%, respectively. A high Ki-67 expression was positively correlated with therapeutic effects. More recently, Huerta *et al.* [37] showed that Ki-67 expression independently predicted the pathologic response to pRCT. These findings should be confirmed.

### 3.5. p21

p21 is a cyclin-dependent kinase whose activation results in cell cycle arrest at the G1- to S-phase transition in mammals [68]. Its expression is predominantly induced by p53, and it is considered a mediator of the tumor-suppressor activity of p53 [69]. However, it also appears to function *via* mechanisms independent of p53 in response to cellular DNA damage [70].

While most of the studies found no correlation between p21 and pCRT in rectal cancer [32,34,39], Sturm *et al.* [31] found a positive correlation between p21 and response to treatment. Tumors with positive pre-therapeutic p21 expression showed a better local tumor response. However, the persistence of highly p21-expressing tumor cells four to six weeks after the completion of a neoadjuvant multimodal therapy had an adverse effect on survival. The persistence of p21-expressing tumor cells may reflect a selection process of resistant tumor cells by the neoadjuvant therapy.

### 3.6. Bax/Bcl-2

Bax and bcl-2 are two proteins involved in the apoptotic/survival pathway. Overexpression of bcl-2 can inhibit cell death in response to many apoptotic signals [26]. On the other hand, loss of bax function in colorectal cancer cells has been linked with resistance to chemotherapeutic agents, whereas induction of bax confers a better chemosensitivity [71,72].

In a study group of 31 patients, 60% of complete responders *versus* 16% of partial responders were bcl-2-positive [67]. Bax and bcl-2 expression as predictive markers of response to pRCT was studied in 130 rectal cancers [32]; bax expression was higher in TRG responders than in TRG non-responders. Moreover, bax expression was higher in complete rather than partial responders (54% *vs.* 29%). This finding may support the notion that apoptosis plays a strategic role in response to pRCT and that bax expression may be a molecular marker of chemoradiosensitivity. These data were confirmed by Huerta *et al.* [37], who found that high bax and low bcl-2 expression predict a good response. The multiple logistic regression analysis reported by Kikuchi *et al.* [43] revealed the bax score as an independent factor, with sensitivity and specificity for predicting responder cases of 82.8 and 83.9%, respectively. However, Moral *et al.* [35] and Lin *et al.* [39] found no correlation between bcl-2 expression and treatment outcome.

### 3.7. Cyclooxygenase-2

Cyclooxygenase (COX)-2 is an enzyme that catalyses the conversion of arachidonic acid to prostaglandins (PGs). Tumor cells can use COX-2 to produce PGs, which protect against radio-induced cell death; thus, it is an important mediator of tumor invasiveness.

Smith *et al.* [67] studied COX-2 expression in 49 rectal cancer patients undergoing pRCT. Patients with overexpression of COX-2 in pre-treatment biopsies were more likely to not respond to pRCT than patients with normal COX-2 expression. Min *et al.* [73] found that COX-2 overexpression was related to poor response to pRCT. This conclusion highlighted a possible role of COX-2 as a predictor of response to pRCT in rectal cancer. However, Debucquoy *et al.* [66] found no correlation between COX-2 up-regulation and response. More studies are required to assess the role of COX-2; it would be interesting to administer a COX-2 inhibitor to patients with COX-2 overexpression and evaluate the impact on response rate.

### 3.8. microRNA

microRNAs (miRNAs) are a group of small, non-coding sequences of RNAs (19 to 22 nucleotides) that play key roles in the regulation of gene expression during crucial cell processes, such as cell differentiation, cell cycle progression, stress response and apoptosis. miRNAs have gained an important role in the control of gene expression, and it is believed that up to 30% of human genes are regulated by miRNAs [74].

Svoboda *et al.* [75] evaluated the expression of miRNAs in tumor biopsies from 35 patients with rectal cancer before pRCT and tumor biopsies from 31 of patients two weeks after starting capecitabine-based pRCT. Increased levels of miR-125b and miR-137 were observed. miR-125b targets Insulin-like Growth Factor Receptor (IGFR)-1, as well as Vascular Endothelial Growth Factor (VEGF) and VEGF receptor (VEGFR). The up-regulation of miR-125b down-regulates its targets and seems to suppress tumor growth and angiogenesis through the insulin/IGFR pathway. miR-137 up-regulation could be important to maintain tumor state [76]. It would be useful to study in depth the role of miRNA expression to identify panels of miRNAs that can predict tumor response to chemo- and/or radiotherapy.

### 3.9. Microarray Studies

Few studies used microarray technology to analyze gene expression profiles in rectal tumors treated with pRCT. Ghadimi *et al.* [77] found 54 genes differently expressed between TNM downstaging responders and non-responders. Using leave-one-out cross-validation, 78% of responders and 86% of non-responders were correctly identified. A validation set of seven different tumor samples hybridized on an alternative microarray platform predicted response in six of seven tumors. Rimkus *et al.* [78] found 42 genes differently expressed between TRG responders and non-responders. Using leave-one-out cross-validation, 71% of responders and 86% of non-responders were correctly predicted. These two studies show the potential of microarrays in predicting response to pRCT; however, there is no concordance with even one gene between the two studies. The complexity and the magnitude of the data are an obstacle to using this technique on a regular basis to predict response to pRCT.

### 3.10. Carcinoembryonic Antigen

In 2006, Park *et al.* [79] investigated the role of serum carcinoembryonic antigen (CEA) level in predicting the response to pRCT in rectal cancer. Upon univariate analysis, CEA level (>5 ng/mL) was significantly associated with both node infiltration and poor response to pCRT. Logistic regression analysis showed that elevated pre-pCRT serum CEA level was the only significant predictor of poor response to pRCT. In 562 patients with non-metastatic rectal cancer, who received pRCT and underwent total mesorectal excision, Das *et al.* [80] confirmed that CEA level (>0.25 ng/mL) predicted tumor pathological response, along with circumferential extent and distance of tumor from the anal verge. Other studies confirmed the predictive role of CEA level. Moureau-Zabotto *et al.* [81] showed that a pretreatment CEA level of <5 ng/mL was independently associated with pCR, and a pretreatment CEA level of <5 ng/mL was significantly associated with tumor downstaging. Perez *et al.* [82] found that a post-pRCT CEA level <5 ng/mL was a favorable prognostic factor for rectal cancer and was associated with increased rates of earlier disease staging and complete tumor regression. Moreover, Moral *et al.* [35] found that patients with lower CEA level (<5 ng/mL) tended to respond better to therapy.

### 3.11. New Potential Circulating Biomarkers

In recent years, researchers have focused on identifying new predictive factors of rectal tumor response to pRCT. Identification of circulating biomarkers is also important for the minimally invasive monitoring of patients. Expression of human telomerase reverse transcriptase (hTERT), the catalytic unit of telomerase, preserves telomere length, thus preventing cell senescence and death, and is essential to the oncogenic process [83,84]. In patients with CRC the level of circulating hTERT mRNA has been correlated with hTERT mRNA level in tumors [85]. Pucciarelli *et al.* [86] showed that the variation of plasma levels of total RNA and hTERT transcripts between pre- and post-pRCT may be potential markers of tumor response in patients receiving pCRT for rectal cancer: In TRG-responders total RNA and hTERT transcript levels significantly decreased, while no differences were observed in non-responders. Moreover, total RNA and hTERT levels were significantly lower in responders than in non-responders.

Agostini *et al.* [87] investigated the dynamics of plasma DNA level and the size distribution of cell-free DNA (cfDNA) before and after neoadjuvant radiochemotherapy as a potential biomarker for the prediction of response to pRCT in patients with locally advanced mid-low rectal cancer. The mean quantity of ALU-247 repeats (tumor cfDNA) was significantly lower post-treatment as compared to pre-treatment in responders, while no differences were found in non-responders. The same findings were obtained using the cfDNA integrity index (pre- and post-treatment values were significantly lower in responders compared with non-responders). These findings suggest that cfDNA may be used as a non-invasive marker of tumor response in patients undergoing neoadjuvant treatment for rectal cancer. However, these findings require validation in larger studies with homogeneous pRCT treatment.

## 4. Discussion

A neoadjuvant radiochemotherapy approach followed by total mesorectal excision has become the standard treatment for patients with locally advanced rectal cancer. Using this approach, results are very encouraging. However, the early and late toxicity and the consequences on bowel function and on some aspects of quality of life related to this approach strongly suggest that patients who will benefit from the treatment need to be carefully selected. Prospective identification of patients who have a higher likelihood of responding to pRCT could be important in decreasing treatment morbidity and improving survival.

The major conclusion of the present study is that there is currently not enough evidence to suggest the clinical application of any biomarker to predict outcome in rectal cancer. The reasons for this conclusion may be related to several factors. The limited number of studies assessing each marker and the small sample size in the majority of studies may have negatively impacted the power of the conclusions. Moreover, most studies are retrospective, further reducing the robustness of results. The most promising biomarkers should be validated prospectively and in large, independent studies involving a large size of patients. Additionally, it is quite difficult to compare studies with different schedules of treatment. Moreover, in several studies, patients received different doses of radiation and drugs. Higher radiation doses and a longer interval between the completion of radiotherapy and the date of surgery may result in higher rates of pCR. Indeed, patients receiving the short-course neoadjuvant therapy (5 Gy /day for 5 days and surgery a week later) very rarely show a pCR, while an interval of more than seven weeks between chemoradiation and surgery was associated with pCR and a low rate of local recurrence [88-90]. Therefore, to avoid biases related to the treatment, biomarkers of tumor response should be evaluated in studies using homogeneous pRCT regimens. A further weakness of the studies concerns the methods used to define the tumor response. Tumor response has been evaluated in different ways. The most used, TRG, was used differently in terms of scoring and grouping. Furthermore, the effect on TNM downstaging is based on a comparison between the preoperative clinical stage and pathologic stage. The accuracy of preoperative clinical staging is as poor [10,91] as it is in the post-pCRT clinical staging [10]. Therefore, the validation of several biomarkers may be impaired by the poor sensitivity of the TNM downstaging in defining the tumor response. Tumor response should be simply better for small tumors that have been erroneously staged as advanced tumors using the current imaging modalities of CT, MRI, and TRUS. Furthermore, increasing evidence shows that the way tumors respond to radiation is dynamic, with up- and down- regulation of genes evident even in the early stages of treatment [92-94]. This potentially means that response may be more accurately predicted by additional studies of the tumor tissue after the initial treatment cycles rather than only analyzing pre-treatment tissues [95]. Currently, there is a growing interest in the role of 18F-FDG PET for the prediction of tumor response to therapy. Recent studies are very heterogeneous with respect to the method applied for PET quantification, the evaluation interval, the metabolic response criteria and the clinical endpoints [96]. Moreover, novel imaging strategies, including functional imaging, will increase accuracy for the overall assessment of response and will be useful in predicting overall outcome [97,98].

In conclusion, to date, none of the investigated biomarkers can be useful in clinical practice. New technologies and approaches, such as microarrays, miRNA analyses and searches for circulating molecules, will provide new potential markers. It is important that old and new biomarkers will be studied in larger, prospective trials that have the same staging, treatment and response criteria.

## Figures and Tables

**Table 1.** p53 expression and prediction of response to neoadjuvant radiochemotherapy in locally advanced rectal cancer patients.

**Author**	**Pts. n.**	**Method**	**Treatment**	**Endpoint**	**Comment**
Chang [32]	130	IHC	50.4 Gy 5-FU + LV	TRG	No correlation
Sturm [31]	66	IHC	45 Gy Heat shock hyperthermia 5-FU + LV	TNM downstaging	No correlation
Lin [39]	70	IHC	45 Gy ± 5-FU	TNM downstaging	Lack of p53 expression associated with poor response
Bertolini [33]	91	IHC	50 Gy 5-FU	TRG TNM downstaging DFS/OS	No correlation
Kudrimoti [38]	17	IHC	50.4–59.4 Gy 5-FU	pCR vs. PR	No correlation
Jakob [36]	22	IHC	50.4 Gy 5-FU	TRG	No correlation
Terzi [30]	37	IHC	45 Gy 5-FU	TRG TNM downstaging	No correlation
Negri [34]	57	IHC	40-45 Gy ±5-FU + Oxa	pCR	No correlation
Moral [35]	39	IHC	42 Gy 5-FU + LV	TNM downstaging	No correlation
Huerta [37]	38	IHC	50.4 G Capecitabine	Tumor size	No correlation

IHC: immunohistochemistry; 5-FU: 5-fluoruracil; LV: leucovorin; Oxa: oxaliplatin; TRG: tumor regression grade; TNM: tumor node metastasis staging; DFS: disease-free survival; OS: overall survival; pCR: pathologic complete response; PR: partial pathologic response.

**Table 2.** TS expression and prediction of response to neoadjuvant radiochemotherapy in locally advanced rectal cancer patients.

**Author**	**Pts. n.**	**Method**	**Treatment**	**Endpoint**	**Comment**
Terrazzino [52]	125	PCR/DNA	45–50.4 Gy5- FU or FU + LV or FU + Oxa or FU + Carboplatin	TRG	No correlation
Bertolini [33]	91	IHC	50 Gy 5-FU	TRG TNM downstaging OS/DFS	No correlation
Spindler [49]	60	PCR/DNA	65 Gy UFT + LV	TRG	TS 2R/2R associated with tumor regression
Jakob [36]	22	PCR/RNA	50.4 Gy 5-FU	TRG	Low TS expression associated with tumor regression
Stoehlmacher [47]	40	PCR/DNA PCR/RNA	50.4 Gy 5-FU	TRG	TS 3′-UTR 6 bp deletion slightly associated with tumor response
Negri [34]	57	IHC	40-45 Gy ± 5-FU + Oxa	pCR	High TS expression associated with higher rate of response
Kikuchi [43]	60	IHC	45 Gy Irinotecan	TRG	Higher TS expression associated with better response
Carlomagno [42]	46	IHC	45 Gy Capecitabine + Oxa	TRG	Low TS expression associated with low response
Hur [50]	44	IHC PCR/DNA	45 Gy 5-FU	TRG TNM	Low-expression genotypes associated with TNM downstaging; no correlation with TRG
Paez [51]	51	PCR/DNA	45 Gy 5-FU	TRG TNM	Genotype 3R/3R associated with tumor response

TS: thymidylate synthase; PCR: polymerase chain reaction; IHC: immunohistochemistry; 5-FU: 5-floruracil; LV: leucovorin; Oxa: oxaliplatin; UFT: Uftoral; TRG: tumor regression grade; OS: overall survival; DFS: disease free survival; TNM: tumor node metastasis staging; pCR: pathological complete response.

**Table 3.** EGFR expression and prediction of response to neoadjuvant radiochemotherapy in locally advanced rectal cancer patients.

**Author**	**Pts. n.**	**Method**	**Treatment**	**Endpoint**	**Comment**
Giralt [55]	87	IHC	45–50.4 Gy ± 5-FU + LV or UFT + LV	pCR DFS/OS Metastasis-free survival	EGFR expression associated with decreased pCR rate
Kim [54]	183	IHC	50 Gy 5-FU + LV	TRG TNM downstaging	Low EGFR expression associated with TNM downstaging
Spindler [48]	77	PCR/DNA	65 Gy UFT + LV	TRG	EGFR Sp1-216 associated with tumor response
Spindler [49]	60	PCR/DNA	65 Gy UFT + LV	TRG	Combination of TS 2R/2R and EGF 61A/G or EGFR Sp1-216T associated with tumor regression
Bertolini [33]	91	IHC	50 Gy 5-FU	TRG TNM downstaging DFS/OS	No correlation
Toiyama [56]	40	PCR/RNA	20 Gy 5-FU + UFT	TNM Grading	Low EGFR expression associated with high response rate
Bengala [**58**]	39	IHC, FISH PCR/DNA	50.4 Gy 5-FU + Cetuximab	TRG	High EGFR GCN and wild-type KRAS associated with response to treatment
Debucquoy [**57**]	41	IHC	50.4 Gy FU + LV	TNM downstaging TRG	No correlation
Bengala [59]	146	ICH, FISH PCR/DNA	50 Gy 5-FU ± Oxa + Capecitabine	TRG DFS/OS	No association of EGFR GCN and KRAS with TRG and OS

EGFR: epidermal growth factor receptor; ICH: imunohistochemistry; PCR: polymerase chain reaction; FISH: fluorescence in situ hybridization; 5-FU: 5-floruracil; LV: leucovorin; Oxa: oxaliplatin; UFT: Uftoral; pCR: complete pathological response; DFS: disease-free survival; OS: overall survival; TRG: tumor regression grade; TNM: tumor node metastasis staging; TS: thymidylate synthase; EGF: epidermal growth factor; EGFR GCN: EGFR gene copy number.
